# Efficacy of combined axitinib with dacarbazine in a B16F1 melanoma xenograft model

**DOI:** 10.3892/ol.2013.1345

**Published:** 2013-05-14

**Authors:** XIAO-HUA ZHANG, EN-QI QIAO, ZHENZHEN GAO, HUA-QING YUAN, PEI-FEN CAI, XIAO-MIN LI, YAN-HONG GU

**Affiliations:** 1 Department of Clinical Oncology, The First Affiliated Hospital of Nanjing Medical University, Nanjing, Jiangsu 210029;; 2 Department of General Surgery and Research Center for Clinical Oncology, Affiliated Jiangsu Cancer Hospital of Nanjing Medical University, Nanjing, Jiangsu 210009;; 3 Department of Emergency, The First People’s Hospital of Lianyungang, Lianyungang, Jiangsu 222000, P.R. China

**Keywords:** axitinib, dacarbazine, melanoma

## Abstract

In this study, we evaluated the efficacy and intestinal side effects of the selective inhibitor of vascular endothelial growth factor (VEGF) receptors, axitinib and/or dacarbazine (DTIC), in a B16F1 melanoma xenograft model. C57BL/6 mice were subcutaneously inoculated with B16F1 melanoma cells. The study was randomized into four groups receiving either 0.5% carboxyl methylcellulose, DTIC, axitinib or a combination of DTIC and axitinib. When the experimental period was complete, the tumor tissues from each mouse were excised, photographed and weighed. The tumor and intestinal tissues were harvested with 4% paraformaldehyde, and paraffin-embedded sections were prepared for hematoxylin and eosin staining, immunohistochemical staining (with antibody specific to proliferating cell nuclear antibody) and terminal deoxynucleotidyl-transferase-mediated dUTP nick end labeling assays. The expression of the VEGF and matrix metalloproteinase 9 genes was analyzed using real-time polymerase chain reaction. No significant benefit to treatment with a combination of axitinib and DTIC, as opposed to axitinib alone, was observed; however, the combined treatment did not enhance the level of enteritis compared with that observed in the axitinb group. In addition, axitinib, as a single agent, demonstrated an improved treatment efficacy compared with DTIC. Therefore, axitinib represents a potential novel, efficient and safe anticancer agent, suggesting a possible use for this schedule in treating melanomas that are less sensitive to DTIC.

## Introduction

The incidence of cutaneous melanoma is annually increasing worldwide (1). Surgical therapy is often curative with a good prognosis in early-stage melanoma, while metastatic melanoma has a median survival time of only 6–9 months (2). Dacarbazine (DTIC) alkylating agent is a long-established treatment for metastatic melanoma, and is considered to be the standard by which other therapeutic agents are evaluated (3). However, DTIC, as a single agent, has no evident effect on overall survival (3). In a previous study, no single agents or combination of agents yielded a significant improvement in clinical responses or overall survival, compared with DTIC monotherapy (4). Therefore, the development of a novel treatment approach is required. Axitinib (AG-013736) is a vascular endothelial growth factor receptor (VEGFR) tyrosine kinase inhibitor (TKI) with greater receptor specificity than that of other VEGFR TKIs being developed for the treatment of a number of malignancies. Axitinib was demonstrated to be efficacious as a single treatment agent in patients with renal cell cancer, who were no longer responding to first-line TKI therapy, in a phase III trial (5). Axitinib, as a single agent, has demonstrated promising activity in a number of tumor types (6–9). It inhibited the development of spontaneous lymphatic and lung metastases in murine melanoma models, and enhanced the protection associated with bevacizumab therapy when used in a combination protocol against orthotopic M24met xenografts (10). A multicenter phase II study has demonstrated the effect of axitinib as a single-agent therapy in patients with metastatic melanoma (11). Axitinib treatment resulted in an 18.8% objective response rate, comparing favorably with standard melanoma therapies (12). The present study indicated potential new approaches to addressing the clinical application of combined treatments of chemotherapeutic agents and VEGFR inhibitors, with regard to the relative antitumour activity. Certain combination therapies have been demonstrated to increase the antitumor activity of axitinib *in vivo.* Such therapies include metronomic and standard doses of cyclophosphamide (13,14), gemcitabine, docetaxel and carboplatin (10), which have been successfully used *in vivo* in human pancreas, breast and ovarian cancer xenografts.

No preclinical data are currently available regarding combined axitinib and DTIC treatment. The purpose of the current study was to investigate whether there was a synergistic antitumor effect between axitinib and DTIC *in vivo*.

## Materials and methods

### Cell lines and reagents

The B16F1 cell lines were purchased from the Shanghai Institute of Biochemistry and Cell Biology (Shanghai, China). The cells were cultured in Dulbecco’s modified Eagle’s medium (DMEM) supplemented with 100 U/ml penicillin, 100 *μ*g/ml streptomycin and 10% fetal calf serum (Gibco, Carlsbad, CA, USA), in a humidified 5% (v/v) CO_2_ atmosphere at 37°C. All other chemicals were purchased from Sigma-Aldrich (St. Louis, MO, USA).

### Animals

Female C57BL/6 mice (age, 6–8 weeks; weight, 18–22 g) were provided by the Model Animal Genetics Research Center of Nanjing University (Nanjing, China) and group-housed at a specific pathogen-free facility under controlled temperature (22±2°C) and a 12-h light-dark cycle. The mice were allowed to acclimate to these conditions for ∼1 week prior to inclusion in the experiments. For each group of experiments, the mice were matched by body weight and tumor size. The animal welfare and experimental procedures were in accordance with the Guide of the Care and Use of Laboratory Animals (The Ministry of Science and Technology of China, 2006), and the related ethical regulations of Nanjing University. Efforts were conducted to minimize the animals’ suffering and to reduce the number of animals used.

### Treatment agents

Axitinib (purity, >99%) and DTIC (purity, >99%) were purchased from Hubei Xinyinhe Chemical Engineering Company (Wuhan, China). The axitinib, a white to light-yellow crystalline powder, was stored at −20°C and protected from light. It was formulated in a homogeneous suspension of 0.5% carboxyl methylcellulose (CMC; ICN Pharmaceuticals France SA, Orsay, France) at 4°C, while protected from light. The DTIC, a white crystalline powder, was stored at 4°C and protected from light. It was dissolved in 0.9% sodium chloride solution to a specific concentration, and intraperitoneally injected into the mice with melanoma.

### Tumor therapy model

Mice were subcutaneously inoculated (in the right flank) with 5×10^5^ B16F1 melanoma cells suspended in 100 *μ*l phosphate-buffered saline (PBS). The average tumor volume was 60–100 mm^3^ prior to randomization of the study into four groups (10 mice per group) on the day of initial drug treatment. Vehicle, DTIC, axitinib or a simultaneous combination of DTIC and axitinib were administered. Mice in the control group were intraperitoneally (i.p.) injected with PBS daily for 5 days, while recieving 0.5% CMC twice a day for two weeks, in equivalent quantities and with the same schedule as the treatment groups. In the DTIC group, DITC (80 mg/kg, i.p.) was administered daily for 5 days. In the axitinib group, axitinib was orally administered via a gastric tube twice a day for 14 days, at a dose of 25 mg/kg body weight and a volume of 5 *μ*l/g. A simultaneous combination of axitinib and DTIC was administered to the combination treatment group, in accordance with the aforementioned schedules. The experimental period ended 14 days after the last administration of treatment. Animal body weights were monitored, and the length and width of the tumors were measured each day throughout the study using calipers. The tumor volume was defined as follows: Volume (mm^3^) = length (mm) × width^2^ (mm^2^) × π/6. When the experiment was terminated, the mice were sacrificed by cervical dislocation. The tumor tissue from each mouse was excised, photographed and weighed. To establish the impact on survival, in an additional experiment (8 mice per group), the tumor volume data was collected and analyzed with a one-way analysis of variance (ANOVA) test (GraphPad Prism; GraphPad Software, Inc., La Jolla, CA, USA). Each treatment group was further compared with the vehicle control group using a Dunnett’s test, to assess the statistical significance of the differences. P<0.05 and P<0.01 were considered to indicate a statistically significant difference.

### Real time quantitative polymerase chain reaction (PCR)

Total RNA was isolated from 50 mg tumor tissue using the RNeasy extraction kit (Qiagen, Hilden, Germany). The expression of each target gene was normalized to that of the housekeeping gene, glyceraldehyde-3-phosphate dehydrogenase (GAPDH). Gene-specific PCR was conducted with AmpliTaq DNA Polymerase (Applied Biosystems, Carlsbad, CA, USA), and primer pair amplifications were performed over 35–40 cycles. All primers were obtained from GenScript (Nanjing, China). The cycling conditions were as follows: Initial denaturation at 94°C for 5 min, denaturation at 94°C for 30 sec, annealing at 58–61°C for 30 sec, and elongation at 72°C for 45 sec, with a total of 35–40 cycles. The primer sequences used were as follows: Sense: 5′-CGCGAGTCTGTGTTTTTGCA-3′ and antisense: 5′-CAGAGCGGAGAAAGCATTTGT-3′ for VEGF; and sense: 5′-CATCGAACTTCGACACTGAC-3′ and antisense: 5′-AGCCACGACCATACAGATAC-3′ for MMP9. The band intensities for gene-specific products were then normalized to GAPDH, which served as the endogenous housekeeping gene between samples. The normalized transcript levels are presented as the mean fold change (± standard deviation) compared with the baseline values for each specific group.

### Hematoxylin and eosin (H&E) staining, immunohistochemistry (IHC) and terminal deoxynucleotidyl-transferase-mediated dUTP nick end labeling (TUNEL) analysis of apoptotic cells

When the experiments were complete, the tumor tissues were harvested with 4% paraformaldehyde, and paraffin-embedded sections were prepared for H&E staining, TUNEL assays and IHC (with antibody specific to proliferating cell nuclear antibody, PCNA). The tumor tissues were cut into 5-*μ*m sections, deparaffinized in xylene and serially dehydrated in decreasing concentrations of ethanol. Sections were stained with H&E and examined under a light microscope. The TUNEL assay was performed using the In Situ Apoptosis Detection kit (Beyotime, Nanjing, China). Following incubation with proteinase K (20 *μ*g/ml) at 25°C for 30 min, the TUNEL reaction mixture, containing BrdUTP, terminal deoxynucleotidyl transferase and reaction buffer, was added to the slides, which were incubated in a humidified chamber at 37°C for 60 sec. The slides were then rinsed and incubated with a fluorescein isothiocyanate-labeled anti-BrdU monoclonal antibody at room temperature for 30 min. The reaction was visualized by fluorescence microscopy. Following heat-induced antigen retrieval and the addition of blocking serum (Power Block 1:10; BioGenex, San Ramon, CA, USA) with anti-mouse PCNA antibody (Santa Cruz Biotechnology, Inc., Santa Cruz, CA, USA) and rabbit anti-rat secondary antibody (1:50; BD Pharmingen, San Diego, CA, USA), 4-*μ*m sections were incubated. The fluorescent signals were detected with a mercury lamp (Olympus U-RFL-T BX51, Nanjing Ology Instrument Co., Ltd., Nanjing, China) and analyzed by Image-Pro Plus 6.0 (Media Cybernetics, Inc., Bethesda, MD, USA).

### Statistical analysis

All experiments were repeated three times with similar outcomes. The statistical significance of the differences was evaluated by a one-way ANOVA followed by a Dunnett’s test. P<0.05, P<0.01 and P<0.001 were considered to indicate a statistically significant difference. Data presented in the figures represent the mean ± standard error.

## Results

### Axitinib, alone and in combination with DTIC, demonstrates significant antitumor activity against melanoma flank xenografts

**Figure 1.**
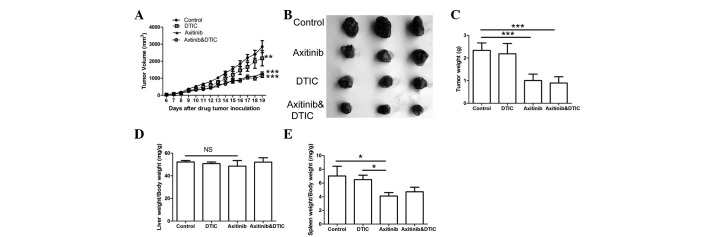
Antiproliferative effects of the vehicle, dacarbazine (DTIC), axitinib, and axitinib and DTIC combination treatments in mice inoculated with melanoma xenografts. (A) The tumor volume was monitored and recorded. ^**^P<0.01 and ^***^P<0.001, compared with the control group. (B) Representative photographs of the tumor sections are shown. (C) Tumors excised at day 14 were weighed. (D) Liver and (E) spleen indices. The experiments were performed three times with identical results. Data are expressed as the mean ± SEM. The number of mice in each group is 10. ^*^P<0.05, ^**^P<0.01 and ^***^P<0.001. NS, not significant.

To evaluate the antitumor effects of the combination therapy of axitinib and DTIC *in vivo*, we demonstrated the efficacy of axitinib and/or DTIC treatment in melanoma xenograft models. When the average tumor volume reached 60–100 mm^3^, we divided the mice into four groups; vehicle (0.5% CMC), DTIC (80 mg/kg), axitinib (25 mg/kg), or a simultaneous combination of DTIC and axitinib were administered. The length and width of the tumors were measured daily throughout the study, using calipers. The experimental period ended 14 days after the last administration of treatment. The weights of the tumor, liver and spleen were monitored. The results indicated that the axitinib and combination treatment groups demonstrated significantly decreased tumor growth and weights compared with the control group (P<0.001, Fig. 1A-C); however, there was no significant difference in such characteristics between the axitinib and combination treatment groups. No significant difference in the liver weights among all groups was identified (Fig. 1D). We also found that the combination treatment group did not exhibit a significant difference in the weight of the spleen compared with the control or DTIC treatment groups; however, a significant difference in spleen index was demonstrated between the axitinib and control groups (P<0.05) (Fig. 1E).

### Treatment reduces tumor cell proliferation, decreases the area of tumor necrosis and increases apoptosis

**Figure 2.**
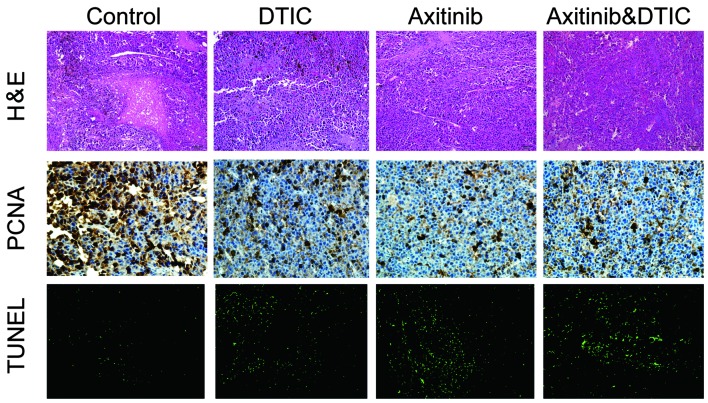
Following the administration of axitinib, dacarbazine (DTIC), a combination of axitinib and dacarbazine, or vehicle, tumor tissue sections were obtained from mice that had previously been inoculated with melanoma xenografts. The sections were stained with hematoxylin and eosin (H&E), antibody specific to proliferating cell nuclear antibody (PCNA) and terminal deoxynucleotidyl-transferase-mediated dUTP nick end labeling (TUNEL), to detect changes in the levels of necrosis, proliferation and apoptosis, respectively. Original magnification, ×200.

We investigated whether DTIC, axitinib or a simultaneous combination of DTIC and axitinib had an impact on tumor cell necrosis, proliferation and apoptosis, by measuring H&E staining, IHC staining (of PCNA) and TUNEL in the mice, respectively. We observed that all drug treatment groups exhibited decreased areas of tumor necrosis, reduced tumor proliferation and enhanced tumor cell apoptosis, compared with the control group (Fig. 2). However, there was no clear difference in these factors between the combination and axitinib treatment groups.

### Axitinib, alone and in combination with DTIC, reduces meta-tasis-related factors and prolongs lifespan in mice

**Figure 3.**
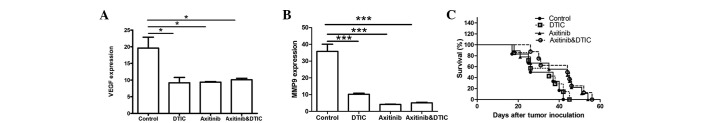
Axitinib, alone and in combination with dacarbazine (DTIC), affects metastasis-related factors and lifespan in mice inoculated with melanoma xeno-grafts. (A and B) Effect of DTIC, axitinib or a simultaneous combination of DTIC and axitinib on VEGF and MMP9 gene expression by quantitative polymerase chain reaction analysis. Columns, mean; bars, SEM. The experiments were performed twice, with similar results. ^*^P<0.05 and ^***^P<0.001. (C) Axitinib, alone and in combination with DTIC, had a more prolonged effect than the vehicle or DTIC. C57BL/6 mice were allowed to live until their spontaneous death. The survival rates of mice inoculated with melanoma xenografts in an additional experiment (eight mice per group) were recorded and shown as Kaplan-Meier curves.

VEGF and MMP9 genes are associated with tumor progression. Therefore, we set out to investigate whether DTIC, axitinib, or a combination of DTIC and axitinib may reduce the expression of these two genes while preserving antitumor activity. Our results indicated that all drug treatment groups exhibited significantly decreased expression of VEGF and MMP9 compared with the control group (Fig. 3A and B); however, no statistically significant differences among the groups were identified. Moreover, we investigated whether axitinib/DTIC prolongs life span in melanoma mice in a further experiment (eight mice per group). C57BL/6 mice were allowed to live until their spontaneous death. We found that treatment with axitinib, alone and in combination with DTIC, had a more prolonged effect than that of the vehicle or DTIC (Fig. 3C). Treatment with axitinib, alone and in combination with DTIC, resulted in a prolonged life span (median survival time, 44.5 or 44 days, respectively), compared with that of the vehicle (31.5 days) or DTIC (35 days) treatment. However, no significant difference between axitinib and combined axitinib/DTIC in prolonging life span was observed.

### Intestinal side effects

**Figure 4.**
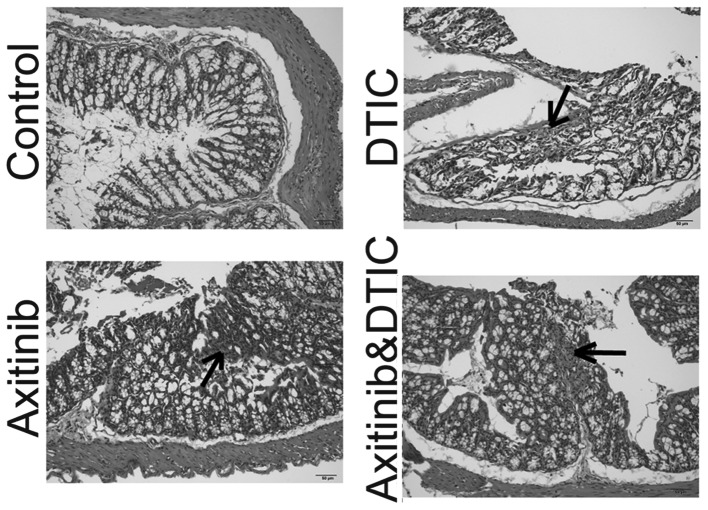
Hisptopathological changes induced by the vehicle, dacarbazine (DTIC), axitinib, or a simultaneous combination of DTIC and axitinib in mice inoculated with melanoma xenografts. The intestine samples were fixed with formalin, embedded in paraffin and sectioned. The hisptopathological sections were stained with hematoxylin and eosin. Original magnification, ×200.

Enteritis is a common side effect of chemotherapy in the clinic (15). Such intestinal side effects often interfere with the implementation of chemotherapy and may reduce the efficacy of drugs. We set out to investigate whether axitinib and DTIC combination therapy enhanced intestinal inflammation, by measuring H&E staining. We identified that DTIC, axitinib, and a combination of DTIC and axitinib treatment induced enteritis. The staining of the enteritis caused by DTIC appeared lighter than that of the axitinib group, and the axitinib and DTIC combination group. In addition, axitinib in combination with DTIC did not enhance the level of enteritis compared with that induced by axitinib alone (Fig. 4).

## Discussion

Malignant melanoma is well-known for its rapid progression and poor response to currently applied treatments. There were ∼68,130 new cases of melanoma diagnosed in 2010, with an estimated 8,700 fatalities caused by this disease in the United States (16). DTIC is the most commonly used therapy for advanced/metastatic melanoma. In a previous study, no single agent or combination of agents yielded a significant improvement in clinical responses and overall survival compared with DTIC monotherapy (4). Consequently, the development of new therapeutic agents for melanoma with greater efficiency is required. Axitinib is a small-molecule oral TKI that is a relatively selective and potent inhibitor of VEGFR−1, −2 and −3 at clinically achievable doses, compared with numerous other anti-angiogenic agents in its class. It has demonstrated antitumor effects *in vivo,* mainly due to the anti-angiogenic property of the molecule, as demonstrated by IHC (17,18). It has been used as a single agent in certain phase II/III studies in various malignancies, such as renal cancer (5,6), non-small cell lung cancer (8), thyroid carcinoma (7) and metastatic melanoma (10). As new anti-angiogenic drugs enter the clinic for cancer treatment, and as an increasing number of candidates progress through preclinical and clinical development, it is important to obtain an improved understanding of the effects of such drugs on tumor blood vessel patency, and their potential interactions with traditional cancer chemotherapies. Studies have combined axitinib with chemotherapeutic agents in treating a number of malignancies, such as pancreatic (19,20), breast (21) and metastatic colorectal (22) cancer; however, there is no preclinical data currently available regarding treatment with a combination of axitinib and DTIC.

In our study, we demonstrated that the axitinib and DTIC treatment combination did not significantly decrease the growth or weight of the tumors in the mice, compared with that of axitinib treatment alone. This also indicated that axitinib, as single agent, may show a greater efficacy compared with DTIC in decreasing the tumor volume and weight. However, the spleens of mice treated with axitinib demonstrated significant weight loss compared with the control group, while those of the DTIC and combination groups did not. This implies that axitinib may induce splenic toxicity. Certain chemotherapeutic agents are able to kill target cells primarily by inducing apoptosis. Our study demonstrated that DTIC, axitinib, and the combination of DTIC and axitinib significantly decreased the area of tumor necrosis (the premature death of cells in living tissue), reduced tumor proliferation and enhanced tumor cell apoptosis, compared with that of the control group. However, no significant difference was identified between the axitinib and combination treatment groups. MMP9 and VEGF were correlated with tumor progression, stimulating tumor growth and metastasis. MMP9 is specifically induced in premetastatic lung endothelial cells and macrophages by distant primary tumors via VEGFR-1/Flt-1 TK, and it significantly promotes lung metastasis (23). We investigated whether the treatment groups demonstrated significantly downregulated VEGF and MMP9 mRNA expression compared with the control group; however, no statistically significant differences between the groups were observed. Previously, no single agents or combination of agents have been identified to exert a significant improvement on overall survival compared with DTIC monotherapy (4). However, in the present study, we observed that treatment with the axitinib/DITC combination, and with axitinib alone, resulted in a prolonged lifespan (median survival time, 44.5 and 44 days, respectively), compared with that of treatment with vehicle or DTIC (31.5 and 35 days, respectively). No significant difference was identified between axitinib in combination with DTIC and axitinib alone in prolonging lifespan. Enteritis is a common adverse effect of chemotherapy; it is a frequently observed side effect of VEGFR TKIs in the clinic (24). It often interferes with the implementation of chemotherapy, and may reduce the effectiveness of drugs. We found that all drug treatments with DTIC, axitinib or a combination of DTIC and axitinib caused enteritis. The staining of the enteritis caused by DTIC appeared lighter than that in the axitinib or axitinib combined with DTIC groups, while the axitinib combined with DTIC group did not increase the level of enteritis compared with the axitinib-treated group.

In conclusion, to the best of our knowledge, our study demonstrated for the first time that the effect of combined axitinib and DTIC *in vivo* was not superior to treatment with axitinib alone. The results also demonstrated that axitinib, as a single agent, may possess a greater treatment efficacy than DTIC. This indicated that axitinib may represent a promising novel, efficient and safe anticancer treatment agent, suggesting a possible use for this schedule in treating melanomas that are less sensitive to DTIC.
